# Detection of Powdery Mildew in Two Winter Wheat Plant Densities and Prediction of Grain Yield Using Canopy Hyperspectral Reflectance

**DOI:** 10.1371/journal.pone.0121462

**Published:** 2015-03-27

**Authors:** Xueren Cao, Yong Luo, Yilin Zhou, Jieru Fan, Xiangming Xu, Jonathan S. West, Xiayu Duan, Dengfa Cheng

**Affiliations:** 1 State Key Laboratory for Biology of Plant Disease and Insect Pests, Institute of Plant Protection, Chinese Academy of Agricultural Sciences, Beijing, China; 2 Key Laboratory of Integrated Pest Management on Tropical Crops, Ministry of Agriculture, Environment and Plant Protection Institute, Chinese Academy of Tropical Agricultural Sciences, Haikou, China; 3 Department of Plant Pathology, China Agricultural University, Beijing, China; 4 East Malling Research, East Malling, Kent, United Kingdom; 5 Rothamsted Research, Harpenden, United Kingdom; Leibniz-Institute of Vegetable and Ornamental Crops, GERMANY

## Abstract

To determine the influence of plant density and powdery mildew infection of winter wheat and to predict grain yield, hyperspectral canopy reflectance of winter wheat was measured for two plant densities at Feekes growth stage (GS) 10.5.3, 10.5.4, and 11.1 in the 2009–2010 and 2010–2011 seasons. Reflectance in near infrared (NIR) regions was significantly correlated with disease index at GS 10.5.3, 10.5.4, and 11.1 at two plant densities in both seasons. For the two plant densities, the area of the red edge peak (*Σdr*_680–760 nm_), difference vegetation index (DVI), and triangular vegetation index (TVI) were significantly correlated negatively with disease index at three GSs in two seasons. Compared with other parameters *Σdr*_680–760 nm_ was the most sensitive parameter for detecting powdery mildew. Linear regression models relating mildew severity to *Σdr*_680–760 nm_ were constructed at three GSs in two seasons for the two plant densities, demonstrating no significant difference in the slope estimates between the two plant densities at three GSs. *Σdr*_680–760 nm_ was correlated with grain yield at three GSs in two seasons. The accuracies of partial least square regression (PLSR) models were consistently higher than those of models based on *Σdr*_680760 nm_ for disease index and grain yield. PLSR can, therefore, provide more accurate estimation of disease index of wheat powdery mildew and grain yield using canopy reflectance.

## Introduction

Wheat powdery mildew, caused by the obligate fungi *Blumeria graminis* f. sp. *tritici* (*Bgt*), is a worldwide destructive foliar disease of wheat. Since the late 1970s, the occurrence of wheat powdery mildew has tended to be more severe in China [1]. Management strategies for wheat powdery mildew are mainly based on host resistance and fungicides [2]. However, the rapid emergence of new virulent races of the pathogen often causes varieties to lose their resistance in a relatively short period of time. Application of fungicides is still essential for disease management [3]. It is, therefore, important to accurately monitor the occurrence and severity of the disease in order to time fungicide applications.

The conventional method for disease severity assessment in the field mainly relies on direct observation [4]. This method is often time consuming and in addition it may vary considerably among assessors. As an alternative method, remote sensing can be used to non-destructively assess plant diseases rapidly, repeatedly over a large area without physical contact with sampling units.

The application of remote sensing in agriculture typically involves measuring reflectance of electromagnetic radiation in the visible (390 to 770 nm), near-infrared (NIR, 770 to 1,300 nm), or middle-infrared (1,300 to 2,500 nm) ranges using spectrometers [5]. Hyperspectral sensors measure reflectance continuously as a series of narrow wavelength bands. To provide pertinent information on plant biophysical parameters (i.e., chlorophyll content) or to correct for background interference from soil or the atmosphere, hyperspectral reflectance data are usually converted to vegetation indices, where two or more important wavebands are mathematically combined [6]. Remote sensing has been applied for detection of numerous crop diseases [7–14] and it has been reviewed in various publications [4–5], [15–18].

Also remote sensing has shown its potential application in detecting cereal powdery mildew. Lorenzen and Jensen [19] found a change of reflectance in the visible spectra of barley leaves infected with powdery mildew. The most sensitive response of reflectance to leaf damage caused by wheat powdery mildew infections was within the range of 490–780 nm [20]. Two QuickBird data and one airborne hyper-spectral HyMap datasets were used to examine the potential use of multi-spectral remote sensing in wheat powdery mildew detection [21]. PLSR (partial least square regression) and FLDA (the fisher linear discriminant analysis) were efficient in estimating the severity of winter wheat powdery mildew on leaves using the selected spectral features [22]. Cao *et al* [23] also used canopy hyperspectral reflectance to detect wheat powdery mildew in two winter wheat cultivars, and found that spectral indices at growth stage (GS) 10.5.3 (flowering over at base of ear), 10.5.4 (flowering over, kernel watery ripe) and 11.1 (milky ripe) were significantly correlated negatively with disease index. Zhang *et al* [24] used moderate resolution multi-temporal satellite imagery to monitor powdery mildew of winter wheat.

It was reported that grain yield can be estimated using spectral reflectance during different crop growth stages [25–27]. Also there were some reports on the relationships between canopy reflectance and yield when diseases occurred, including peanut-late leaf spot [28], alfalfa-leaf spot [29], and Asian soybean rust [30]. These reports indicated the potential of canopy reflectance in grain yield prediction.

All of these studies on wheat powdery mildew detection or monitoring were focused on wheat planted at the same densities, however, previous work has shown that reflectance at NIR ranges was highly correlated with plant density and vigor [31–32]. Relationships between nitrogen nutrition of wheat canopies and the spectral indices were affected by water supply and plant density [33]. Canopy reflectance of barley was affected by above ground plant density [34]. Few studies have been made on using canopy reflectance to predict wheat grain yield when powdery mildew is present.

This study was conducted to (i) develop models relating spectral indices to the severity of wheat powdery mildew at two pant densities; (ii) to compare the performance of spectral indices and PLSR for estimating powdery mildew of winter wheat; and (iii) to predict grain yield of wheat using canopy reflectance.

## Materials and Methods

### 2.1. Experimental plot and inoculation

Experiments were established in the same field at the Langfang Experimental Station, Institute of Plant Protection, Chinese Academy of Agricultural Sciences (39°30′42″N, 116°36′07″E) in Hebei Province, in 2009–2010 and 2010–2011 growing seasons. A double cropping system (two crops per year) was employed at the site since 2002, with winter wheat followed by soybean. Flooding irrigation was performed six times to promote mildew development during the season and carbamide was applied as the basic fertilizer. Weeds were controlled by hand weeding. Winter wheat cultivar, Jingshuang 16, highly susceptible to powdery mildew, was used in the experiments. Seeds were sown in rows 0.25 m apart on 6 October for both seasons. Two seeding rates (60 kg seed ha-1, plant density 1 and 120 kg seed ha-1, plant density 2) were used.

Mildew inoculum was prepared in a greenhouse. Seedlings of cultivar Jingshuang 16 sown in 10 cm pots (about 50 seeds in a pot) were inoculated by dusting *Bgt* spores on the leaves ten days after sowing. The inoculated plants were incubated under 18°C for 7 days. The pots with infected seedlings were placed in the center of the experimental plots on 2 April 2010 and 25 March 2011 to initiate disease development. In order to attain a range of disease severities, fungicide triadimefon was applied at one of the six concentrations (300, 240, 120, 60, 30, 15 g active ingredient ha^-1^). One only spray per year was applied. Control plots were sprayed with water on 13 April 2010 and 15 April 2011, respectively. The experiments were performed with randomized block design. Total of 21 plots, 5 m long and 4 m wide each, were planted with three replicates for each density.

### 2.2. Disease assessment

Since spectral indices at growth stage (GS) 10.5.3, 10.5.4 and 11.1 were reported to correlate negatively with wheat powdery mildew severity [23], powdery mildew was assessed at these three GSs [35]. Within each plot, disease severities were assessed at five positions (four at the corners and one at the centre). At each position, 20 plants (within 10 cm diameter) were randomly selected to record disease severity of powdery mildew by using a 0-to-9 scale [36–37]. 0: free from infection, 1: a few isolated lesions on only the lowest leaves, 3: light infections on the lower third of the plant with the lower most leaves infected at moderate to severe levels, 5: severe infection on lower leaves with light to moderate infection on the middle leaves, 7: severe infections on both lower and middle leaves with some infection on the flag leaf as well, and 9: severe infection on all leaves with spikes infected as well. So for every plot, a total of 100 plants were recorded. The average disease index (DI) for a plot was calculated as:

$$\text{DI}=\frac{0\times n0+1\times n1+......+9\times n9}{9\times (n0+n1+......+n9)}\times 100$$

Where n0, n1……n9 are the number of plants with severity of 0, 1…9, respectively.

### 2.3. Reflectance measurements

Canopy percentage reflectance data were acquired with an ASD Field Spec Pro spectrometer (Analytical Spectral Devices, Boulder, CO, USA) when mildew was assessed. This spectrometer has a sampling interval of 1.4 nm for the 350 to 1,050 nm region of the electromagnetic spectrum (3 nm spectral resolution) and 2 nm for the 1,050 to 2,500 nm region (10-nm spectral resolution), with a field of view of 25°. Both 1.4 nm and 2 nm sampling intervals are automatically interpolated to 1 nm intervals by the instrument. The sensor, facing downwards at the centre of the plot, was positioned 0.5 m from the top of the wheat canopy, covering a 22.16 cm diameter field of view. Measurements were taken on clear, sunny days between 10:00 h and 14:00 h (Beijing time, GMT + 8:00). The instrument was referenced to a calibrated spectral on a white reflectance panel about every 15 min while readings were obtained, allowing readings from different assessment dates to be compared. At each sampling date, 20 different positions near the centre were used and the average value was calculated for further analysis.

### 2.4. Grain yield data

Grain yield data used in the study were from a previously published paper [38]. In the paper, grain yield in each plot at plant density 2 was recorded (kg ha^−1^).

### 2. 5. Data analysis

The reflectance spectra were analyzed using ViewSpecPro software (Analytical Spectral Devices, Inc.). The averaged raw reflectance was smoothed by the Savitzky–Golay filter in Origin Pro Version 8 (OriginLab Corporation, Northampton, MA, USA) with a frame size of 15 data points (2nd degree polynomial). This filter was adjusted for the local signal-to-noise ratio in order to smooth the target spectrum point by point [39]. After smoothing, the first derivative spectra of each plot were computed with an interval of 1 nm.

To identify optimal indices for assessing wheat powdery mildew severity, the smoothed raw reflectance data were combined into various narrowband and wideband spectral indices (SIs) (Table 1). A total of 17 spectral features indices were used, including four derived from reflectance of broad-band, 10 from reflectance of single-band, and three red edge parameters from the first derivative reflectance. It had been shown that there were significant differences in spectral reflectance indices of wheat at different GSs [27] [40]. Therefore, the relationships between disease index and each reflectance range or vegetation index as well as red edge parameters at each GS were assessed using correlation analysis (SAS Institute Inc., Cary, NC). Variables with high correlation were included in regression analysis in which the two planting densities and three GSs were treated as two factors. The effect of two planting densities and three GSs on the relationship of disease index with spectral reflectance was assessed by the interaction term of these two factors with model parameters (intercept and slope). The significance of slopes and intercepts was tested by dropping terms from the model and assessing the change in the residual sum of squares using an *F*-test using PROC GLM procedure of SAS (SAS Institute Inc, Cary, NC, USA, 1996). Also the relationships between grain yield and spectral indices were assessed using correlation analysis. Variables with high correlation were selected to develop regression models to predict grain yield.

**Table 1:** Vegetation indices used in this study and their method of calculation.

spectral indices	Definition	Description or formula	Literatures
DVI	difference vegetation index	R_NIR_—R_R_	[41]
RVI	ratio vegetation index	R_NIR_ / R_R_	[42]
NDVI	normalized difference vegetation index	(R_NIR_—R_R_)/ (R_NIR_ + R_R_)	[43]
GNDVI	Green normalized differencevegetation index	(R_NIR_—R_G_)/ (R_NIR_ + R_G_)	[44]
NBNDVI	Narrow-band normalized difference vegetation index	(R_850_—R_680_)/(R_850_ + R_680_)	[45]
NRI	Nitrogen reflectance index	(R_570_—R_670_)/(R_570_ + R_670_)	[46]
TVI	Triangular vegetation index	0.5[120(R_750_—R_550_)- 200(R_670_—R_550_)]	[47]
PRI	Photochemical/Physiological Reflectance Index	(R_531_—R_570_)/(R_531_ + R_570_)	[48]
PhRI	The Physiological Reflectance Index	(R_550_—R_531_)/(R_550_ + R_531_)	[48]
TCARI	The transformed chlorophyllabsorption and reflectance index	3[(R_700_—R_670_)- 0.2(R_700_—R_550_)(R_700_/R_670_)]	[49]
MCARI	Modified chlorophyll absorption ratio index	[(R_701_—R_671_)- 0.2(R_701_—R_549_)]/(R_701_/R_671_)	[50]
RVSI	Red-Edge Vegetation Stress Index	[(R_712_+R_752_)/2]—R_732_	[51]
PSRI	Plant Senescence Reflectance Index	(R_680_—R_500_)/R_750_	[52]
ARI	Anthocyanin Reflectance Index	(R_550_)^-1^ - (R_700_)^-1^	[53]
* λ*_*red*_	red edge position	Wavelength position at red edge slope	[54]
*dr*_*red*_	red edge slope	Maximum value of 1^st^ derivative with in red edge	[54]
*Σdr*_680-760 nm_	the area of the red edge peak	the area under the derivative curve in the region of red edge	[54]

R_R_ = Reflectance of red band with the range from 650–680 nm

R_NIR_ = Reflectance of near-infrared band with the range from 780–890 nm

R_G_ = Reflectance of green band with the range from 560–600 nm.

Partial least square regression (PLSR), which may overcome the problems of collinearity and “over-fitting” compared to stepwise multiple linear regression analysis, was used to model the relationship between canopy reflectance spectra (predictor variables) and disease index of powdery mildew (response variable). This method was also used for grain yield prediction. The PLSR modeling was performed using Simca 13.0.3 software (Umetrics, Umeå, Sweden).

The performance of the model was assessed by comparing the differences in the coefficient of determination (R^2^) and the root mean square error (RMSE) of observed DI and predicted DI.

## Results

### 3.1. Disease epidemics in the field

**Fig 1:**
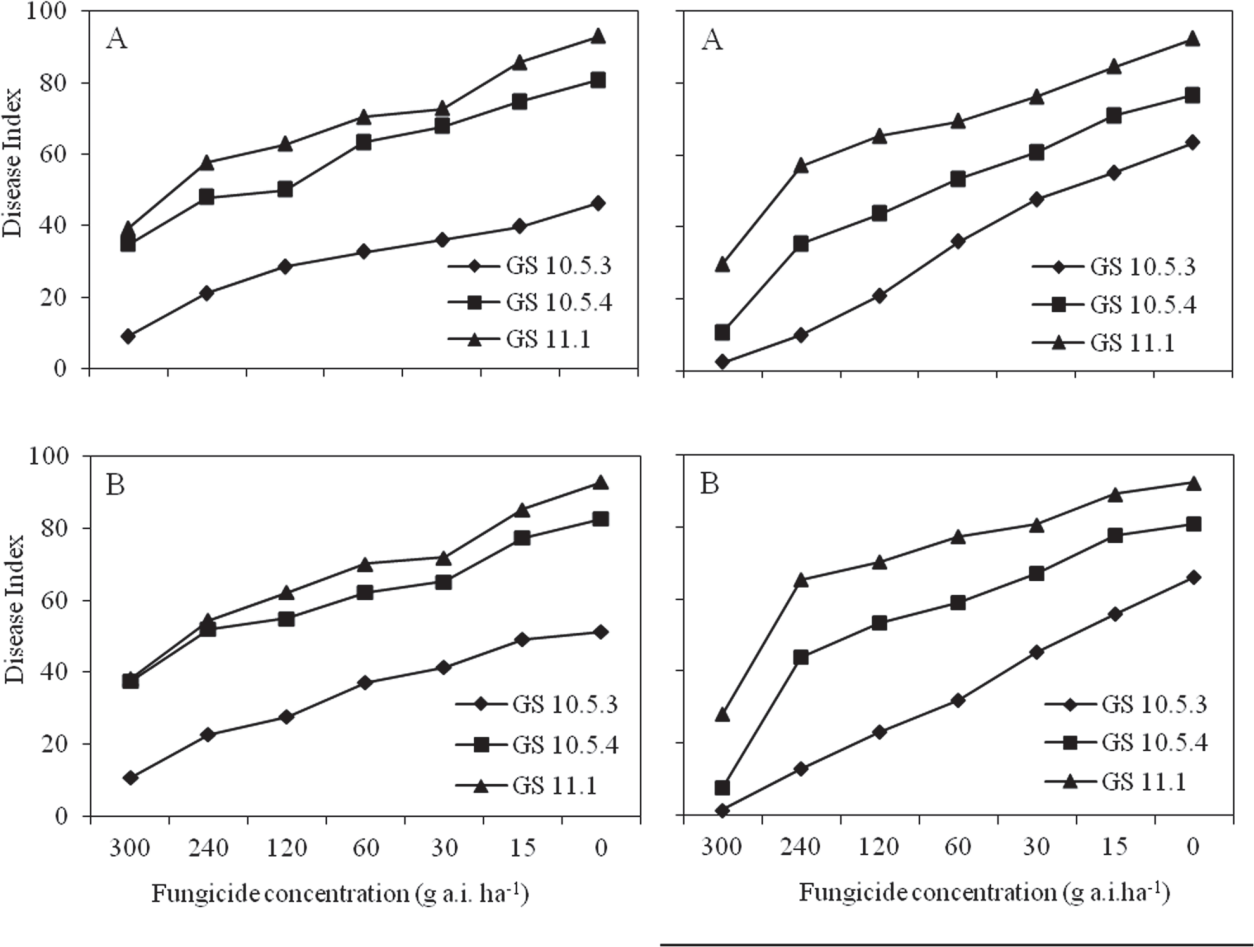
Progress curves of wheat powdery mildew epidemics on plant density 1 (60 kg seed/ha) (A) and density 2 (120 kg seed/ha) (B) with different concentrations of fungicide applications in Langfang City in 2009–2010 and 2010–2011 seasons. Disease severity was assessed (0–9 scale) at GS 10.5.3, 10.5.4 and 11.1.

A wide range of powdery mildew disease indexes was obtained across plots for the two plant densities at each assessment date in two seasons (Fig. 1). The maximum disease index in the control plots reached 90 in both seasons and both plant densities when assessed at GS 11.1. At this time, the lowest disease index in plots receiving the highest concentration of fungicide did not exceed 40 and 30 at the two plant densities in 2009–2010 and 2010–2011 seasons, respectively.

In the 2009–2010 season, disease indexes in plots of plant density 2 (120 kg seed ha^-1^) were slightly higher than those in plots of plant density 1 (60 kg seed ha^-1^) at GS 10.5.3 and 10.5.4. However, at GS11.1, disease index was almost the same at the two densities. In the 2010–2011 season, disease indexes in density 2 were slightly higher than those in plots of density 1 at GS 10.5.3, 10.5.4 and 11.1, except those plots receiving the highest fungicide concentration.

### 3.2. Wheat canopy reflectance and its relationship with disease index

**Fig 2:**
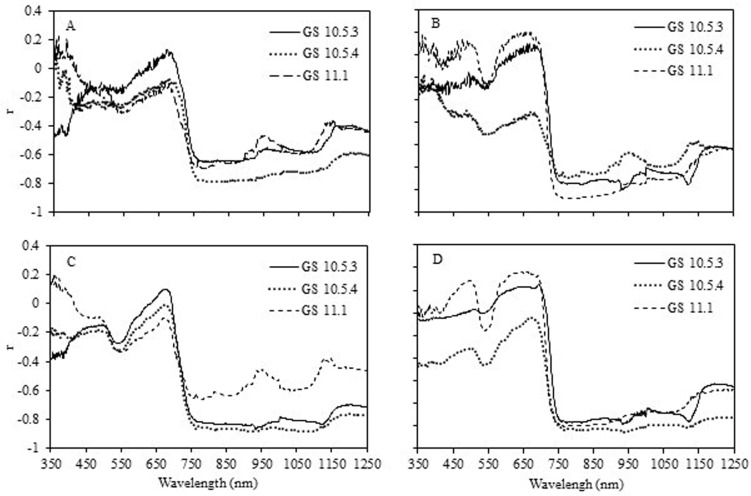
Linear correlation between spectral reflectance and disease indexes of wheat powdery mildew at GS 10.5.3, 10.5.4 and 11.1 in 2009–2010 and 2010–2011 seasons for the plant density 1 (60 kg seed ha^-1^) (A) and density 2 (120 kg seed ha^-1^) (B).

There were significant negative correlations between canopy reflectance in NIR region and disease index at GS 10.5.3, 10.5.4 and 11.1 for two plant densities in the two seasons (Fig. 2). However, the correlations of reflectance in the visible region, especially in the red region, with disease index were not consistent with plant density in the two seasons. In density 2, there were significant correlations between disease index and canopy reflectance in the red region at GS 10.5.3, 10.5.4 and 11.1 in the 2009–2010 season. In contrast, the disease index was positively correlated with canopy reflectance in the red region only at GS 11.1 in 2010–2011. There were no significant correlations between canopy reflectance in the red region and disease index for density 1 in both seasons.

### 3.3. Correlations between spectral indices and disease index

Correlations between spectral indices and disease index are shown in Table 2. *Σdr*_680-760 nm_, DVI and TVI correlated significantly with disease index at both plant densities and in both seasons at GS 10.5.3, 10.5.4 and 11.1; whereas PhRI, PRI, TCARI and MCARI did not or in most cases did not correlate significantly with disease index at the two plant densities and in both seasons at all three GSs. There were significant correlations of RVI, NDVI, GNDVI, NBNDVI, NRI, RSVI, PSRI, ARI, *λ*_*red*_ and *dr*_*red*_ with disease index in all or most cases in plant density 2 in the two seasons. However, these spectral indices did not consistently correlate significantly with disease index for density 1 in both seasons.

**Table 2:** Coefficients of correlation between spectral indices and the index of wheat powdery mildew at different growth stages of wheat at the two plant densities during two seasons.^a^

spectral indices	2009–2010 season								2010–2011 season						
	Density 1				Density 2				Density 1				Density 2		
	10.5.3	10.5.4	11.1		10.5.3	10.5.4	11.1		10.5.3	10.5.4	11.1		10.5.3	10.5.4	11.1
DVI	**−0.65**	−0.75	−0.68		−0.75	−0.69	**−0.87**		**−0.83**	**−0.88**	−0.65		−0.75	−0.82	−0.80
RVI	−0.28	−0.12	−0.50		−0.72	−0.72	−0.83		−0.48	−0.46	−0.64		−0.66	−0.56	−0.79
NDVI	−0.35	−0.21	−0.51		−0.70	−0.74	−0.83		−0.50	−0.48	−0.63		−0.64	−0.58	−0.71
GNDVI	−0.30	−0.16	−0.48		−0.69	−0.58	−0.82		−0.47	−0.44	−0.64		−0.67	−0.56	−0.75
NBNDVI	−0.35	−0.22	−0.54		−0.72	−0.74	−0.82		−0.50	−0.48	−0.62		−0.63	−0.57	−0.70
NRI	−0.36	−0.24	−0.42		−0.64	−0.65	−0.55		−0.47	−0.51	−0.37		−0.48	−0.46	−0.54
TVI	−0.62	−0.74	−0.70		−0.75	−0.67	−0.86		−0.78	−0.84	−0.66		−0.74	−0.78	−0.78
PRI	−0.34	−0.28	−0.05		−0.74	−0.75	−0.46		−0.46	−0.41	0.01		−0.77	−0.47	−0.53
PhRI	0.22	0.10	−0.20		0.11	0.11	−0.16		−0.25	−0.30	−0.59		0.42	−0.05	−0.26
TCARI	−0.29	−0.28	−0.42		−0.04	−0.08	−0.13		−0.46	−0.52	−0.45		0.08	−0.40	−0.31
MCARI	−0.31	−0.29	−0.41		−0.05	−0.09	−0.20		−0.46	−0.52	−0.45		0.08	−0.40	−0.31
RVSI	−0.49	−0.51	−0.29		−0.73	−0.59	−0.48		−0.49	−0.59	−0.31		−0.75	−0.62	−0.49
PSRI	0.53	0.33	0.19		0.77	**0.75**	0.60		0.50	0.51	0.23		0.65	0.51	0.53
ARI	0.38	0.42	0.17		**0.85**	0.74	0.47		0.54	0.62	0.19		0.75	0.51	0.50
*λ*_*red*_	−0.36	−0.46	0.03		−0.75	−0.37	−0.26		−0.29	−0.49	−0.26		−0.70	−0.56	0.04
*dr*_*red*_	−0.24	−0.62	**−0.77**		−0.30	−0.63	−0.51		0.04	−0.32	−0.73		−0.15	−0.35	−0.41
*Σdr*_680–760 nm_	−0.63	**−0.76**	−0.70		−0.76	−0.68	**−0.87**		−0.79	−0.84	**−0.67**		**−0.76**	**−0.83**	**−0.81**

^a^Plant density 1 and 2 represent 60 and 120 kg seed ha^−1^, respectively.

^b^Values in bold indicated the highest correlation values.

### 3.4. Relating disease index to spectral reflectance

*Σdr*_680-760 nm_ showed an overall higher and consistent correlation with disease index compared to other spectral reflectance indices. Parallel curve analysis showed that there was no significant difference in the slope for the derived linear models between the two plant densities at GS 10.5.3 in each seasons (*P* = 0.39, 0.61 for the two seasons, respectively). There was no significant difference in the slope for the models between the two densities and seasons at GS 10.5.4 and 11.1. There was a significant difference in intercept for the constructed models between the two plant densities and seasons. Therefore, models with the same slope but different intercept were constructed (Table 3). All models explained more than 50% of the total variability in disease index except those of plant density 1 at GS 10.5.3 in the 2009–2010 season and GS 11.1 in the 2010–2011 season.

**Table 3:** Parameter estimates of models, relating disease index of wheat powdery mildew to the area of the red edge peak (*Σdr*_680-760 nm_) at three growth stages (GS) at the two plant densities in 2009–2010 and 2010–2011 seasons.^a^

Season	Density^b^	10.5.3^c^		10.5.4		11.1	
		a	b	a	b	a	b
2009–2010	1	−451.31±73.89	158.62±24.13	−563.69±86.08	230.46±24.96	−317.30±41.93	163.67±12.02
	2		163.27±27.07		221.90±23.13		163.11±10.63
2010–2011	1	−890.69±115.86	262.94±32.96		213.34±21.30		162.55±9.23
	2		286.54±38.36		204.78±19.46		161.98±7.83

^a^The disease index (DI) of wheat powdery mildew was a function of *Σdr*_680-760 nm_, therefore DI = a×*Σdr*_680-760 nm_ + b.

^b^Plant density 1 and 2 represent 60 and 120 kg seed ha^-1^, respectively.

^c^Growth stage using Feekes scale.

The R^2^ and the relative RMSE values of the models based on *Σdr*_680-760 nm_ and PLSR were summarized in Table 4. The accuracies of the PLSR models were consistently higher than those of models based on *Σdr*_680-760 nm_ except for plant density 2 at GS 10.5.4 in the 2009–2010 season.

**Table 4:** Summary of regression models for predicting disease index of wheat powdery mildew.

Model	2009–10 season								2010–11 season						
	Density 1				Density 2				Density 1				Density 2		
	10.5.3^a^	10.5.4	11.1		10.5.3	10.5.4	11.1		10.5.3	10.5.4	11.1		10.5.3	10.5.4	11.1
*Σdr*_680-760 nm_															
R^2^	0.39	0.57	0.50		0.57	0.52	0.76		0.63	0.71	0.45		0.58	0.63	0.62
RMSE	9.15	10.98	12.11		9.13	17.84	12.26		13.25	19.27	16.84		14.10	16.22	13.96
PLSR															
R^2^	0.41	0.59	0.67		0.66	0.49	0.75		0.71	0.84	0.62		0.65	0.69	0.62
RMSE	8.96	9.54	9.67		7.99	10.29	8.55		11.63	8.54	11.32		12.76	12.87	12.25

^a^Growth stage using Feekes scale.

### 3.5. Correlations between spectral indices and grain yield

All spectral indices except R_R_ and *λ*_*red*_ correlated significantly with yield in both seasons (Table 5). *λ*_*red*_ only had significant correlation with grain yield at GS 10.5.3 in both seasons. All the spectral indices showed low correlation with grain yield at GS 10.5.4 and high correlations at GS 10.5.3 and 11.1. All the spectral indices at GS 10.5.3 in 2009–2010 season and R_NIR_, DVI and *Σdr*_680-760 nm_ at GS 10.5.3 in 2010–2011 season showed higher correlations with grain yield when compared with GS 11.1. Mean indices over the three growth stages always had higher correlations with grain yield than at 10.5.4 and 11.1 in both seasons. The mean indices over the three growth stages provided higher correlations with grain yield compared to GS 10.5.3 in the 2010–2011 season.

**Table 5:** Coefficients of correlation between spectral indices and grain yield at different growth stages of wheat infected by powdery mildew during two seasons.

spectral indices^a^	2009–2010				2010–2011			
	10.5.3 ^b^	10.5.4	11.1	Mean^c^	10.5.3	10.5.4	11.1	Mean
RED	−0.397	−0.395	−0.269	−0.394	−0.301	−0.192	−0.303	−0.341
NIR	0.789	0.533	**0.739**	0.765	0.712	0.551	0.705	0.805
DVI	0.804	0.564	0.728	0.766	0.713	**0.557**	0.709	0.805
RVI	0.688	0.527	0.549	0.661	0.553	0.460	0.611	0.644
NDVI	0.695	**0.570**	0.599	0.676	0.530	0.452	0.572	0.636
*λ*_*red*_	0.655	0.121	−0.112	0.406	0.656	0.288	−0.056	0.519
*dr*_*red*_	0.496	0.407	0.466	0.656	0.081	0.089	0.490	0.185
*Σdr*_680-760 nm_	**0.810**	0.504	0.691	**0.775**	**0.715**	0.552	**0.714**	**0.812**

^a^DVI = difference vegetation index, RVI = ratio vegetation index, NDVI = normalized difference vegetation index, .*λ*_*red*_ = red edge position, *dr*_*red*_ = red edge slope, *Σdr*_680-760 nm_ = the area of the red edge peak.

^b^Growth stage using Feekes scale.

^c^Correlation between yield and mean spectral reflectance indices across three growth stages.

### 3.6. Relating grain yield to spectral reflectance

*Σdr*_680-760 nm_ had higher correlations with grain yield in the three growth stages in the two seasons. Models relating grain yield to *Σdr*_680-760 nm_ at GS 10.5.3, 10.5.4 and 11.1 and the mean *Σdr*_680-760 nm_ over the three growth stages were constructed. There was no significant difference in the slope at every GS and mean value of *Σdr*_680-760 nm_ over the three growth stages between the two seasons; however, there was a significant difference in the intercept between the two seasons. Therefore, models with the same slope but different intercepts were constructed (Table 6). The models accounted for more than 50% of the total variability in grain yield except at GS 10.5.4 (Table 7).

**Table 6:** Parameter estimates of models, relating grain yield of wheat infected by powdery mildew to the area of the red edge peak (*Σdr*_680-760 nm_) in 2009–2010 and 2010–2011 seasons.^a^

Season	10.5.3^b^		10.5.4		11.1		Mean^c^	
	a	b	a	b	a	b	a	b
2009–2010	20147.00±2948.86	−2021.41±764.56	13665.17±3494.70	−271.06±977.08	10774.87±1796.13	283.81±129.57	19750.69±2545.07	−2203.58±720.62
2010–2011		−1246.69±653.17		757.17±815.94		1308.38±532.26		−1127.95±607.35

^a^The grain yield (y) of wheat was a function of *Σdr*_680-760 nm_, therefore y = a×*Σdr*_680-760 nm_ + b.

^b^growth stage using Feekes scale.

^c^Mean spectral reflectance indices across three growth stages.

**Table 7:** Summary of regression models for predicting grain yield of wheat infected by powdery mildew.

Model	2009–10 season					2010–11 season			
	10.5.3^a^	10.5.4	11.1	Mean^b^		10.5.3	10.5.4	11.1	Mean
*Σdr*_680-760 nm_									
R^2^	0.66	0.25	0.48	0.60		0.51	0.31	0.51	0.66
RMSE	260.94	375.39	319.80	286.60		415.06	488.27	415.16	354.13
PLSR									
R^2^	0.67	0.32	0.58	0.69		0.52	0.68	0.64	0.68
RMSE	249.19	355.68	278.58	240.36		402.63	331.14	350.19	330.05

^a^Growth stage using Feekes scale.

^b^Mean spectral reflectance indices across three growth stages.

PLSR models also accounted for more than 50% of the total variability in grain yield except at GS 10.5.4 in the two seasons (Table 7). The accuracies of the PLSR models were consistently higher than those of models based on *Σdr*_680-760 nm_ in both seasons at the three GSs and mean the value over three GSs.

## Discussion

Our results demonstrate that spectral reflectance is useful for estimating disease index of wheat powdery mildew at the two wheat plant densities at GS 10.5.3, 10.5.4 and 11.1. The most sensitive spectral region for powdery mildew was in NIR. This is in accordance with previous studies on rice leaf blight and tomato late blast [12] [55]. Also Cao *et al*. [23] reported that reflectance in NIR regions was significantly correlated with disease index of powdery mildew at GS 10.5.3, 10.5.4 and 11.1 for two winter wheat cultivars.

Not all spectral parameters selected in the study were significantly correlated with disease index at the two plant densities. For SIs calculated based on combination of reflectance of broad-band, DVI had significant correlations with disease index in both seasons for the two plant densities. However, RVI, NDVI and GNDVI were only significantly correlated with disease index at GS 11.1 in plant density 1 in the 2009–2010 season. Although in the 2010–2011 season, RVI, NDVI and GNDVI were significantly correlated with disease index, the correlations were smaller than with DVI, indicating that DVI was more appropriate for disease detection. The mathematical calculation methods of SIs may relate to the variation in performance of the vegetative indices in disease detection. It was reported that RVI and NDVI are sensitive to effects of soil reflectance (brightness), especially at low vegetation cover, whereas DVI perform relatively well at low LAI values, i.e. relatively sparse vegetation cover [56].

For SIs calculated based on the combination of reflectance of single-bands, these SIs without reflectance in NIR wavebands, including PhRI, PRI, TCARI, MCARI, did not have or in most cases did not have significant correlations with disease index of wheat powdery mildew in the two plant densities and two seasons evaluated at GS 10.5.3, 10.5.4 and 11.1. This can be explained by significant negative correlations between canopy reflectance in the NIR region at GS 10.5.3, 10.5.4 and 11.1 for two plant densities in the two seasons. TVI, calculated from the differences in reflectance in different wavelengths, had significant correlation with disease index in the two seasons and densities. This was consistent with DVI, which was also calculated using differences between reflectance in different bands. The performance of NBNDVI and NRI was consistent with NDVI as their calculation method was similar.

For red edge, *dr*_*red*_ and *Σdr*_680-760 nm_ were significantly correlated with disease index of wheat powdery mildew at GS 10.5.3, 10.5.4 and 11.1 in the two seasons at the two plant densities, while these correlations were not consistently significant for the red edge waveband (*λ*_*red*_) at GS 10.5.3, 10.5.4 and 11.1 in the two seasons at the two plant densities. This can be explained by the fact that *λ*_*red*_ is only defined by one wavelength, while both *dr*_*red*_ and *Σdr*_680-760 nm_ are defined by more than one wavelength.

The comparison of disease detection models constructed for two plant densities at GS 10.5.3, 10.5.4 and 11.1 in the two seasons based on *Σdr*_680-760 nm_ showed that there was no significant difference in the slope of the models between the two plant densities in the two seasons, which implied that the rate of change at *Σdr*_680-760 nm_ of a wheat canopy infected by powdery mildew was not influenced by the plant density. The significant difference in the intercept indicated that there were differences between canopy reflectance at different plant densities, which is consistent with previous studies [31–34]. Plant density influenced wheat physiology, which in turn influenced canopy reflectance [57]. Also plant density influences disease severity. The commonly held view is that disease decreases with reduced plant density [58]. However, disease severity of barley powdery mildew in susceptible cultivars increased with decreasing density [59] and stripe rust severity increased with planting density in 1997 but decreased with planting density in 1998 [60].

Grain yield of wheat can be assessed using canopy reflectance when powdery mildew occurred. Spectral indices at GS 10.5.3 had higher correlations or almost equal correlations with grain yield when compared with GS 10.5.4 and 11.1. This may be because the best indicator for yield estimation was disease severity at GS 10.5.3 [38]. The mean indices over the three growth stages provided higher correlations with grain yield compared to individual growth stage except GS 10.5.3 in the 2009–2010 season. This was similar to previous studies, which reported that correlations of the mean estimates of the spectral reflectance indices across growth stages with yield were higher compared to any individual growth stage [26–27]. This was probably because that the mean indices across growth stages provide more information compared to single individual growth stages. Canopy reflectance, especially spectral indices at GS 10.5.3, can be used for grain yield estimation, which is very useful. Not only can it save time and labor compared with conventional methods, but also it can estimate yield at early growth stages.

PLSR can provide more accurate estimation of DI and grain yield than models based on *Σdr*_680-760 nm_. A great advantage of PLSR over a traditional regression method is its capability not only in lowering dimensionality of the raw data but retaining the majority of variance contained in the raw data. Also the PLSR provides a regression model in which the entire spectral dataset is taken into account.

Although remote sensing of wheat powdery mildew is a potential alternative for detecting disease, rather than visual assessment of plants, further work is necessary before the method can be adopted for practical use. For example, nutrient deficiencies and other diseases (i.e. rusts and leaf blotch) cause wheat foliage to become chlorotic. Although it was reported that three sugar beet diseases, *Cercospora* leaf spot, powdery mildew and rust can be differentiated using spectral vegetation indices [11], and Yuan *et al*. [61] illustrated the potential use of hyperspectral information in discriminating yellow rust, powdery mildew and wheat aphid infestation in winter wheat at the leaf level, further research is needed to determine whether powdery mildew has a unique spectral signature at the canopy level, which can be used to discriminate it from other foliar problems (biotic and abiotic) that may cause similar responses in fields.
